# Learning from public health and hospital resilience to the SARS-CoV-2 pandemic: protocol for a multiple case study (Brazil, Canada, China, France, Japan, and Mali)

**DOI:** 10.1186/s12961-021-00707-z

**Published:** 2021-05-06

**Authors:** Valéry Ridde, Lara Gautier, Christian Dagenais, Fanny Chabrol, Renyou Hou, Emmanuel Bonnet, Pierre-Marie David, Patrick Cloos, Arnaud Duhoux, Jean-Christophe Lucet, Lola Traverson, Sydia Rosana de Araujo Oliveira, Gisele Cazarin, Nathan Peiffer-Smadja, Laurence Touré, Abdourahmane Coulibaly, Ayako Honda, Shinichiro Noda, Toyomitsu Tamura, Hiroko Baba, Haruka Kodoi, Kate Zinszer

**Affiliations:** 1 Centre Population et Développement (Ceped), Institut de recherche pour le développement (IRD) Université de Paris, ERL INSERM SAGESUD, Paris, France; 2 School of Public Health, University of Montreal, Montréal, Québec Canada; 3 Centre de recherche en santé publique (CRePS), Université de Montréal et CIUSSS du Centre-Sud-de-l’Île-de-Montréal, Montréal, Quebec Canada; 4 Faculté des arts et des sciences, University of Montreal, Montréal, Québec Canada; 5 Laboratory of Ethnology and Comparative Sociology, Université Paris Nanterre/CNRS, Paris, France; 6 UMR 215 Prodig, CNRS, Université Paris 1 Panthéon-Sorbonne, AgroParisTech, Institut de recherche pour le développement (IRD), Aubervilliers, France; 7 Faculté de Pharmacie, Université de Montréal, Montréal, Canada; 8 Pole 1 de recherche sur la transformation des pratiques cliniques et organisationnelles, CISSS de Laval, Montréal, Canada; 9 Centre de recherche en santé publique (CRePS), Université de Montréal, Montréal, Québec Canada; 10 Faculté des sciences infirmières, Université de Montréal, Montréal, Québec Canada; 11 AP-HP, Bichat-Claude Bernard Hospital, Paris, France; 12 IAME, INSERM, Université de Paris, Paris, France; 13 Institut Aggeu Magalhães, Oswaldo Cruz Fondacion, Recife, Brazil; 14 National Institute for Health Research Health Protection Research Unit in Healthcare Associated Infections and Antimicrobial Resistance, Imperial College London, London, UK; 15 MISELI, Bamako, Mali; 16 Research Center for Health Policy and Economics, Hitotsubashi Institute for Advanced Study, Hitotsubashi University, Tokyo, Japan; 17 Bureau of International Health Cooperation, National Center for Global Health and Medicine, Tokyo, Japan; 18 Center Hospital of the National Center for Global Health and Medicine, Tokyo, Japan

**Keywords:** Hospital, COVID-19, Resilience, Equity, Public health, Design, Lessons learned

## Abstract

**Background:**

All prevention efforts currently being implemented for COVID-19 are aimed at reducing the burden on strained health systems and human resources. There has been little research conducted to understand how SARS-CoV-2 has affected health care systems and professionals in terms of their work. Finding effective ways to share the knowledge and insight between countries, including lessons learned, is paramount to the international containment and management of the COVID-19 pandemic. The aim of this project is to compare the pandemic response to COVID-19 in Brazil, Canada, China, France, Japan, and Mali. This comparison will be used to identify strengths and weaknesses in the response, including challenges for health professionals and health systems.

**Methods:**

We will use a multiple case study approach with multiple levels of nested analysis. We have chosen these countries as they represent different continents and different stages of the pandemic. We will focus on several major hospitals and two public health interventions (contact tracing and testing). It will employ a multidisciplinary research approach that will use qualitative data through observations, document analysis, and interviews, as well as quantitative data based on disease surveillance data and other publicly available data. Given that the methodological approaches of the project will be largely qualitative, the ethical risks are minimal. For the quantitative component, the data being used will be made publicly available.

**Discussion:**

We will deliver lessons learned based on a rigorous process and on strong evidence to enable operational-level insight for national and international stakeholders.

## Background

The current approach to controlling COVID-19 pandemic has largely been a strategy aimed to flatten the epidemic curve and lower peak morbidity and mortality [1]. Reducing the intensity of COVID-19 transmission is crucial to avoid overloading health systems and to allow for a more manageable increase and treatment of hospitalized and severe patients. The resilience of health systems, including public health, in response to COVID-19 is under question [2], including in high-income countries such as the USA [3], Spain [3], Taiwan [4], and Italy [5]. At different points during the pandemic, health systems have been unable to meet the laboratory testing and other supply chain demands such as personal protective equipment. Contact tracing has overwhelmed public health departments, often with less than an optimal time delay [6]. In Italy, guidelines were issued for patient selection for intensive care, restricting it to those that stand to benefit the most [7], and globally, there have been bed shortages in intensive care units (ICUs) [8]. In resource-limited settings, such as in Africa or South America, the low performance and resilience of health systems is alarming [9–11].

Several studies have shown that during the COVID-19 pandemic, social adversities such as poor living and working conditions have accumulated for certain social categories [12]. Thus, if policies including public health measures do not take into account various precarious sociodemographic situations (e.g., migrant status, children, language, low income, overcrowded housing, and inability to isolate oneself or the difficulty of protecting oneself), they may contribute to accentuating social adversities and their deleterious effects, whether or not directly related to the transmission of the virus [13–15]. Therefore, to mitigate COVID-19 pandemic social impacts, public health practices must adapt to living environments.

Coordinated and collaborative evidence-based responses are critical for the successful control of a public health emergency and to maintain health system functioning. The many unknowns of COVID-19 have made the response efforts difficult and variable [7, 16], while it is known that improving equitable access to COVID-19 interventions would be a vital step in reducing disease propagation [13, 14]. As stated in the early stages of the pandemic by the Global Research Forum for COVID-19, there is an urgent need to understand the resiliency of health systems in the context of pandemic planning and response [17]. The preconditions of context have significant impact on the resiliency of health systems faced with the COVID-19 crisis [9, 18]. The need to integrate social sciences, health staff, and system resilience into the pandemic response was also identified as one of the priorities [19, 20]. How different hospitals in different countries respond during this pandemic in their preparation and implementation is essential to study and understand [21]. Regarding public health measures, it is vital to understand how social factors were (or not) taken into account in planning COVID-19 interventions.

## Methods

### Research objectives

The aim of this project is to compare the pandemic response to COVID-19 in locations of Brazil, Canada, China, France, Japan, and Mali during the first and second waves of the pandemic. This comparison will be used to identify strengths and weaknesses in the response, including challenges for health professionals and health systems. The research questions are:

- Q1. How was the response planned, organized, and implemented in different COVID-19 referral hospitals?
- Q2. What disruptions were encountered, what strategies were adopted, and what is the resiliency of professionals and hospitals?
- Q3. How were social and health inequalities considered in the design and planning of public health interventions to prevent the spread of SARS-CoV-2?
- Q4. What collective and practical lessons learned from the COVID-19 crisis can be developed for better preparation and response in the future?
- Q5. What are the factors that facilitated or hindered the dissemination and the use of these lessons learned between the countries?
- Q6. How does the COVID-19 burden differ between each country, and what are the similarities and differences in spatial and temporal trends?

### Research design: multiple case study approach

In the field of health systems research, comparative approaches are recommended [22] and are essential to develop operational, transferable lessons. We will use a multiple case study approach with multiple levels of nested analysis [23]. Each hospital and public health intervention will be considered a single case.

**Fig. 1:**
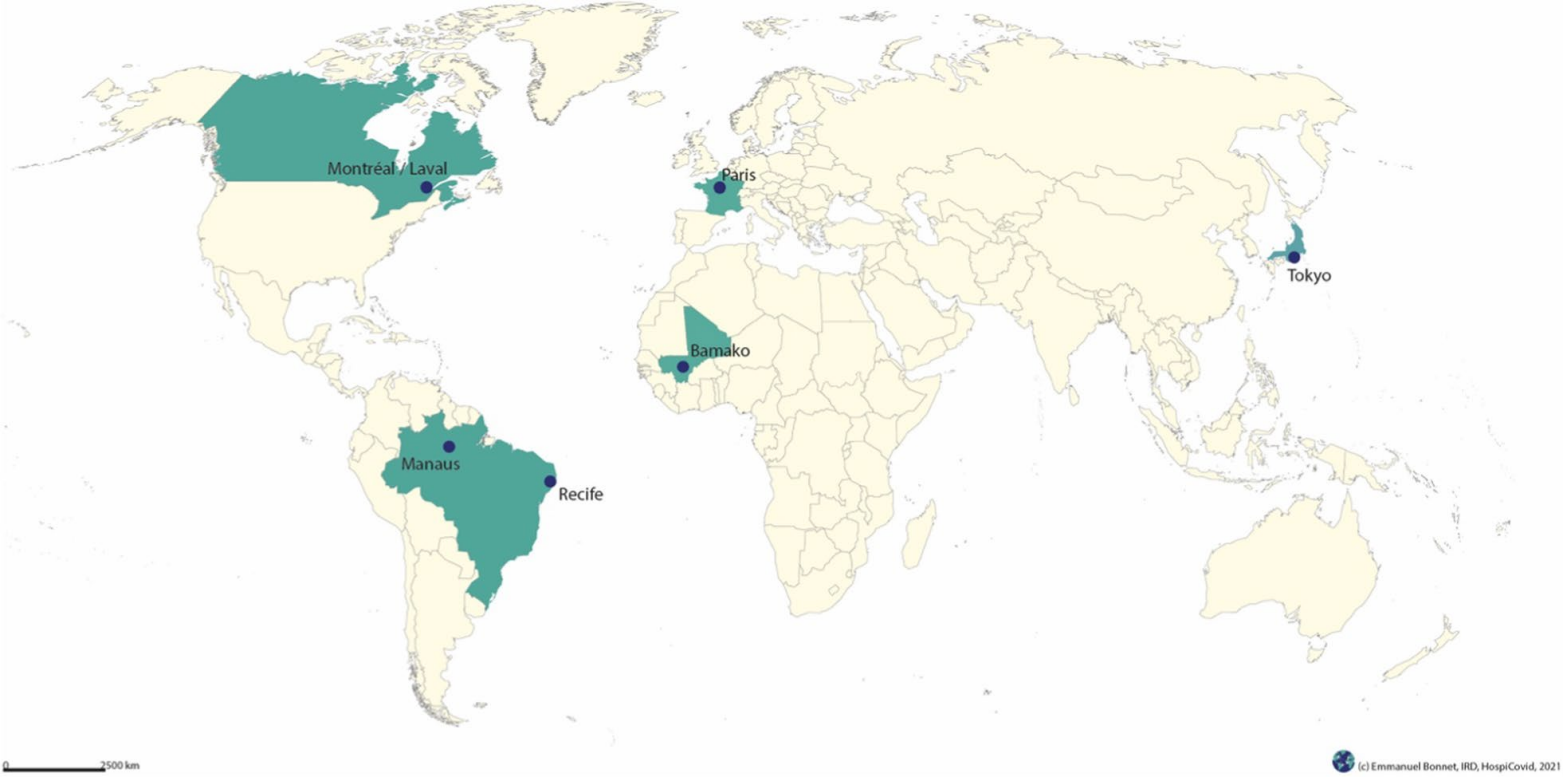
Map of case studies

For the hospital case studies (Q1 and Q2), the analysis will correspond to varying importance of different configurations. The configurations will be identified using a comparative perspective based on the conceptual framework (Fig. 1) [23]. We chose these six countries as they represent the diversity of continents, contexts, and COVID-19 burden, and we have longstanding scientific and practice collaborations. We will focus on eight major hospitals in Recife and Manaus (Brazil), Zhejiang (China), Paris (France), Bamako, (Mali), Montreal and Laval (Canada), and Tokyo (Japan), see Fig. 1.

For the public health case studies (Q3), we will focus on understanding if and how inequalities have been taken into consideration during the planning of two major SARS-CoV-2 infection prevention interventions: contact tracing and testing in the general population. Each intervention at each site will be considered a case study.

For Q4, we will generate high-quality lessons learned (LL) from a systematic approach to collecting, compiling, and analysing data from multiple sources, and reflecting both positive and negative intervention experiences [24]. To develop this process of LL, we conducted a rapid review of the literature, which led to a 10-step guide: (1) identification and mobilization of stakeholders; (2) formulation of the aims of the process; (3) identification of the events targeted to develop the LL; (4) choosing the moment to start the process of developing the LL; (5) selecting the methods; (6) developing interview grids; (7) choosing the data source; (8) data verification and revisions of the aspects to be covered; (9) analysis and formulation of preliminary LL; and (10) verification of the quality of LL.

To share the research results and validate the preliminary LL (step 10), we will organize one deliberative workshop [25] in each country and one international deliberative workshop between the six countries with national institutions and international organizations (WHO, GloPID-R [Global Research Collaboration for Infectious Disease Preparedness], PAHO [Pan American Health Organization], WHO AFRO [WHO Regional Office for Africa], TDR [Special Programme for Research and Training in Tropical Diseases], PHAC [Public Health Agency of Canada], European/Africa CDC [European/Africa Centres for Disease Control and Prevention]). The aim of the workshops will be to discuss the practical implications of the various findings and recommendations, in terms of preparation, interventions, training, and communication. The workshops will be supported by preliminary drafts of policy briefs (PB) [26] to share the research results that led to the preliminary LL, in an accessible format and in multiple languages. This will be part of our an action-oriented approach for decision-makers. Other knowledge transfer (KT) tools (infographics, videos, etc.) will be developed to disseminate these lessons to different audiences, once the LL have been finalized. The project's website will be used for information dissemination and communication (https://u-paris.fr/hospicovid) in English and French.

To evaluate these KT activities (Q5), a mixed-methods design, combining quantitative and qualitative data, will be used.

For Q6, subnational (e.g., provincial, district, or department) COVID-19 portraits will be constructed for each site which will correspond, geographically, to the selected hospitals and public health organizations of Q1–Q3.

#### Data collection

For Q1 and Q2, we will describe how countries have planned, organized, and implemented hospital responses to COVID-19, to describe the resilience of hospitals and their staff. Several empirical data collection techniques will be used (observation, interviews, document analysis). For the observations, the researchers will conduct lengthy observation sessions, when it is safe to do so, over several weeks in some of the hospitals. The aim is to observe the functioning of services, meetings, interaction between professionals, and so on. While these sessions will provide empirical data through systematic note-taking [27], they will also be instrumental for the interviews and developing the interview guide. Qualitative interviews will be conducted with stakeholders using a diversification sampling strategy [28] within each stakeholder group (decision-makers, managers, medical staff, non-medical staff). We anticipate that we will conduct approximately 30 interviews per site/institution, until empirical saturation is reached. The conceptual framework will inform the development of interview guides, which are discussed below. Interview guides will be developed collaboratively and piloted in each jurisdiction prior to use.

For Q3, the two public health measures, contact tracing and SARS-CoV-2 testing, will first be described using the Template for Intervention Description and Replication reporting guideline for population health and policy interventions (TIDier-PHP) for each site [29]. The descriptions will inform the interview guide as well as the conceptual dimensions of the REFLEX-ISS tool, which enables stakeholders to consider the ways that social and health inequalities are taken into account in their interventions [30]. The interview guides will be drafted and tested prior to use for qualitative interviews. These interviews will also be conducted with stakeholders using a diversification sampling strategy [28] within each stakeholder group (decision-makers, managers, public health practitioners). We anticipate that we will conduct approximately 30 interviews per site/institution, until saturation is reached.

For Q4 and Q5, two questionnaires will be used (i) to assess the PB and their use, and (ii) to assess the participants' intention to use the LL. This questionnaire, adapted from the tool developed by Légaré et al.[31], is based on the theory of planned behaviour [32] and Triandis’s theory [33]. Approximately 30 semi-structured interviews will be conducted online 3 months after the national workshops. All members of the research team and at least three key informants in each country will be invited to participate. The interview grid is constructed in part according to the essential organizational components of a deliberative workshop as formulated by Boyko et al [34].

For Q6, publicly available COVID-19 disease surveillance data will be collated for each site along with other data sources such as census (e.g., population, sociodemographic characteristics) and mapping files.

### Conceptual frameworks

Our empirical research for Q1 and Q2 will be supported by an original analytical framework on health system resilience (Box 1).

#### Box 1: Health system resilience definition

**Table:** 

The capacities of a health system faced with shocks, challenges/stress, or destabilizing chronic tensions (unexpected or expected, sudden or subtle, internal or external to the system), to absorb, adapt, and/or transform in order to maintain and/or improve universal access to comprehensive, relevant, and quality health care and services without pushing patients into poverty.	

**Fig. 2:**
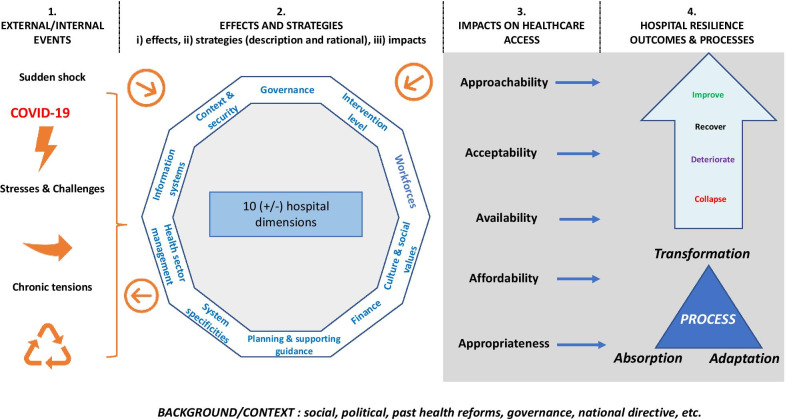
Health system resilience conceptual framework

We will incorporate an analytical framework from the UK Department for International Development [35] as well as aspects of another conceptual frameworks that emphasizes the importance of interactions between the health system and the population in achieving access to care [36–39]. The rationale for our framework (Fig. 2) is that we need to first understand the disruptions encountered by COVID-19, by addressing the question "resilience to what?" The COVID-19 pandemic will be at the core of our analysis, and it represents a series of shocks to the system (1. External/internal events). All types of events (or “situations” to be dealt with) are possible, such as sudden shocks (e.g., COVID-19 pandemic), unique stresses and challenges (e.g., staff and inputs availability), and chronic stresses (e.g., drug shortages). Second, we wish to answer the question "resilience of what?". We wish to uncover the effects (positive or negative) of these events, then the strategies deployed to deal with them (by describing them and explaining their rationale) as well as their impacts on organizational routines and system dimensions (2. Effects and strategies). While the 10 dimensions at the centre of the figure (e.g., governance, human resource, logistics) have been identified in a scoping review, our empirical analysis will be adapted to each hospital’s context [38]. It is important to understand health system “resilience processes” at the individual/team level. We will focus on how managers, health and non-health staff have mobilized resources to respond to the disruptions in the hospital (1. External/internal events). Third, following our definition of resilience (Box 1), we will need to understand how the mobilization (or not) of these strategies in the face of events influences access to health care (3. Impacts on healthcare access) and in particular, the five health system abilities (approachability, acceptability, availability, affordability, appropriateness) of access to care [36]. We will distinguish between these five dimensions for COVID-19 patients from other patients present in the hospitals. We recognize that one of the limitations of our research, due to a lack of resources, will be the difficulty of taking into account the other five demand dimensions of access to care (approachability, acceptability, availability, affordability, appropriateness). Finally, we will examine the combined impacts of these different resilience processes (absorption, adaptation, transformation) and outcomes (improvement, recovery, deterioration, collapse) regarding four different scenarios of hospital resilience (4).

For Q3, we will use the conceptual reflections that guided and informed the development of the REFLEX-ISS tool [40], which are the following: (a) What is the philosophy of social inequalities in health (SIH) in the context at hand? Is there a shared vision of SIH (i.e., a shared vision for the respondent, its institution, and its partners) based on a common analysis of the context and supported by evidence? (b) Was the intervention planning done in consultation with major stakeholders, including community-based organizations (and ensuring their ongoing commitment), and to what extent does the consultation process effectively enact the intersectoral approach at the institutional level (e.g., formalization of consultation spaces, such as setting up partnerships and steering committees) versus only at the individual level (e.g., mobilization of one’s own network of contacts)?

### Analytical approach

All interviews (for Q1, Q2, and Q3) will be transcribed and coded using computer-assisted (or aided) qualitative data processing software guided by the approach and principles of framework analysis, i.e., using a deductive-inductive approach to coding [41].

**Fig. 3:**
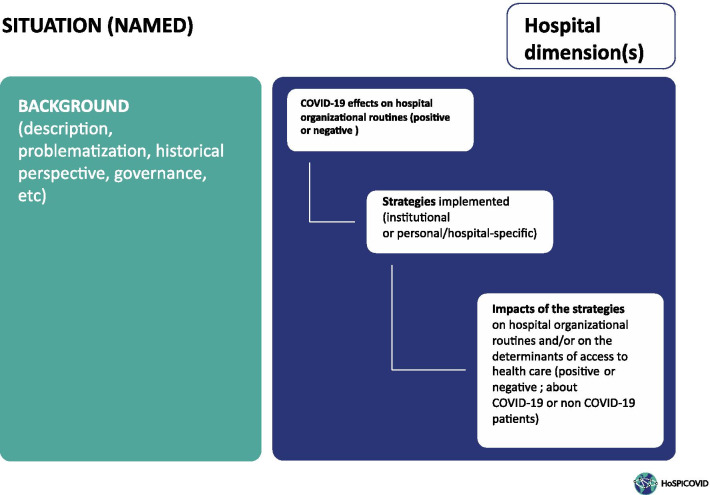
Ideal hospital configuration

From data collected in the hospitals (i.e., interview transcripts and observation notes), descriptive accounts of hospitals’ configurations (10 to 15 for each hospital) will allow us to highlight how adapted dimensions of the framework below are revealed through empirical data. We will uncover the recurrence, according to the specific and historical contexts of hospitals, of configurations between problem situations, the effects on organizational routines, and the strategies deployed to deal with them and the impacts caused (Fig. 3). These configurations will be organized with regard to the dimensions usually analysed in the resilience of health systems using a comparative perspective [38]. The three dimensions of processes from a resilience perspective (effects/strategies/impacts) could give rise to the existence of three types of configurations, for example, reactions (effects/strategies/impacts), anticipations (strategies/impacts before any effects), or inactions (effects but no strategies).

**Fig. 4:**
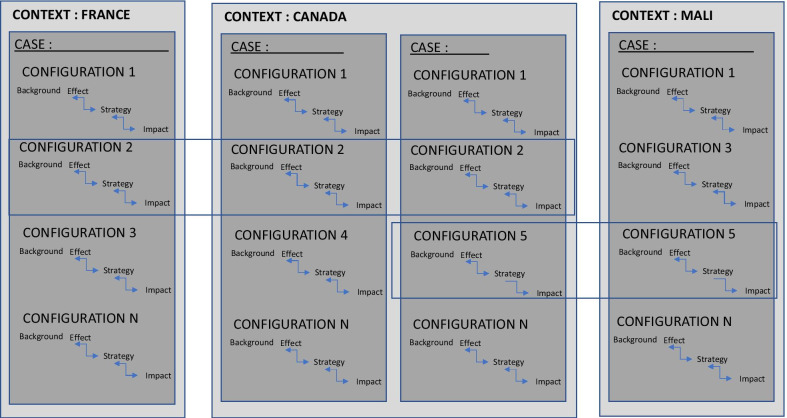
Comparative analysis example

The analytical approach of the multiple case studies will be carried out in two stages. In the first stage, an intra-case analysis will be carried out for each hospital in the study. This analysis will be global and exploratory using a framework analysis approach (Fig. 2) [41]. Throughout the analysis, we will crosscheck and validate the data collected and interpretations with key stakeholders involved in the response. A case study report will be written for each hospital following the plan in Appendix 1. In addition, we will conduct several scoping reviews on the resilience of health systems during the COVID-19 pandemic to ensure that both the historical contexts and up-to-date evidence are incorporated into our analyses. The protocols will available online at (https://www.protocols.io). The second stage of the analysis will be a comparative analysis between the cases, using the configurations as a heuristic tool and to generalize the analysis (Fig. 4).

Based on findings at the hospital level and using our resilience framework (Fig. 2), we will then synthesize the results to make generalizations to similar situations in other locations [23]. This will inform a middle-range theory on the resilience of health care systems and hospitals, i.e., “theories that lie between the minor but necessary working hypotheses that evolve in abundance during day-to-day research and the all-inclusive systematic efforts to develop a unified theory” [42]. The goal is to analyse whether configuration patterns from the different case studies provide the same or different understanding of hospital resilience processes and outcomes. In other words, we will attempt to identify consistencies in the processes and configurations of hospital resilience in the context of a pandemic.

For public health analyses (Q3), we will use the conceptual reflections that guided and/or informed the development of the REFLEX-ISS tool [40].

For Q4 and Q5, due to the small number of workshop participants, quantitative data from the two questionnaires will be subject to descriptive statistics. This will provide a descriptive understanding of the reactions of the participants following their participation in the deliberative workshop as well as their intention to use the knowledge and LL. For the semi-structured interviews, all interviews will be recorded after the consent of the interviewee and then fully transcribed. The transcribed data will then be coded using QDA Miner software and then analysed using a content analysis [43]. During the content analysis, we will seek to understand general trends and discrepancies with an emphasis on comparison between different stakeholders. Triangulation of the results of the qualitative and quantitative analyses will be carried out through a triangulation-convergence approach [44], which involves comparing and contrasting the same object from both sources of data to increase the richness of the interpretation and conclusions.

For Q6, epidemiological curves will be constructed for each site, as will COVID-19 burden maps, at the highest spatial resolution possible (e.g., neighbourhoods). We will create descriptive tables that will include the characteristics of COVID-19 (when possible) as well as timelines of the sequence of events and public health measures for each site.

## Discussion

Our research will provide unique insight into how hospitals in six countries have adapted to the COVID-19 pandemic and how public health interventions have addressed social inequalities in health. Our study is innovative, as it will provide an international comparison of contrasting epidemiological contexts and situations in order to make the knowledge useful to decision-makers through the production of LL. The challenges will be numerous due to the nature of an international research collaboration, particularly in the context of COVID-19, and with a focus on a complex concept such as resilience, with the goal to compare and contrast between very different contexts and cultures. Through our collaborative approach, we anticipate that the challenges will be overcome and that our results will provide relevant information for decision-makers in improving hospital resiliency and for improving the consideration of social and health inequalities in public health interventions.

## Data Availability

The data will be available at https://dataverse.ird.fr.

## Appendix 1. Outline of case study reports for each hospital

**Table:**

**Background** Political, economic, social... situation History of hospital’s reforms and reforms for access to care Description of the national pandemic response and its relations/impacts with hospitals Description of the hospital, how it works (its organization) and its history **Methods** Description of study research methods (if more than one phase, explain the links with epidemic phases) Sampling Descriptive table of stakeholders met and observations made Strategy of analysis **Events** The pandemic in the country and in the hospital Perception of the pandemic by the stakeholders Other events that could potentially affect routines **Configurations** **Dimension 1 (like Governance)** Figure Effects (positive and negative) on organizational routines Strategies (description and justification by the actors) to deal with these effects Impacts (positive and negative) of these strategies on organizational routines **Dimension 2 (like Maintenance)** Figure Effects (positive and negative) on organizational routines Strategies (description and justification by the actors) to deal with these effects Impacts (positive and negative) of these strategies on organizational routines **Dimension N** Figure Effects (positive and negative) on organizational routines Strategies (description and justification by the actors) to deal with these effects Impacts (positive and negative) of these strategies on organizational routines **Impact on access to care** Impact on approachability for COVID-19 patients and others Impact on acceptability for COVID-19 patients and others Impact on availability for COVID-19 patients and others Impact on affordability for COVID-19 patients and others Impact on appropriateness for COVID-19 patients and others **Global analysis** Discussion about the overall resilience of the hospital (or its services) and its evolution over time Discussion about the resilience process in term of absorption, adaptation, transformation Discussion on the presence of configurations (action, reaction, inaction, etc.) Discussion on facilitating and constraining factors of the configurations (what worked well vs what did not work well) Lessons learned (operational recommendations to retain for the future) General discussion Methodological reflections on the challenges of conducting a hospital-based survey in an epidemic situation **General conclusion** **Appendices** Chronology of the events and strategies Interview guides Ethical agreement	

## Abbreviations

COVID-19: Coronavirus disease 2019
European/Africa CDC: European/Africa Centres for Disease Control and Prevention
GloPID-R: Global Research Collaboration for Infectious Disease Preparedness
ICUs: Intensive care units
KT: Knowledge transfer
LL: Lessons learned
PAHO: Pan American Health Organization
PB: Policy briefs
PHAC: Public Health Agency of Canada
Q: Question
REFLEX-ISS: Reflection on social inequalities in health
SARS-CoV-2: Severe acute respiratory syndrome coronavirus 2
SIH: Social inequalities in health
TDR: Special Programme for Research and Training in Tropical Diseases
TIDier-PHP: Template for Intervention Description and Replication reporting guideline for population health and policy interventions
WHO AFRO: WHO Regional Office for Africa
